# The OXR domain defines a conserved family of eukaryotic oxidation resistance proteins

**DOI:** 10.1186/1471-2121-8-13

**Published:** 2007-03-28

**Authors:** Mathieu Durand, Adrianne Kolpak, Timothy Farrell, Nathan A Elliott, Wenlin Shao, Myles Brown, Michael R Volkert

**Affiliations:** 1 Department of Molecular Genetics and Microbiology, University of Massachusetts Medical School, Worcester, Massachusetts 01655, USA; 2 Department of Chemistry and Biology, Université du Québec à Trois-Rivières, C.P. 500 Trois-Rivières, Québec, Canada; 3 Division of Molecular and Cellular Oncology, Department of Medical Oncology, Dana-Farber Cancer Institute, 44 Binney Street, Boston, Massachusetts 02115, USA

## Abstract

**Background:**

The NCOA7 gene product is an estrogen receptor associated protein that is highly similar to the human OXR1 gene product, which functions in oxidation resistance. OXR genes are conserved among all sequenced eukaryotes from yeast to humans. In this study we examine if NCOA7 has an oxidation resistance function similar to that demonstrated for OXR1. We also examine NCOA7 expression in response to oxidative stress and its subcellular localization in human cells, comparing these properties with those of OXR1.

**Results:**

We find that NCOA7, like OXR1 can suppress the oxidative mutator phenotype when expressed in an *E. coli *strain that exhibits an oxidation specific mutator phenotype. Moreover, NCOA7's oxidation resistance function requires expression of only its carboxyl-terminal domain and is similar in this regard to OXR1. We find that, in human cells, NCOA7 is constitutively expressed and is not induced by oxidative stress and appears to localize to the nucleus following estradiol stimulation. These properties of NCOA7 are in striking contrast to those of OXR1, which is induced by oxidative stress, localizes to mitochondria, and appears to be excluded, or largely absent from nuclei.

**Conclusion:**

NCOA7 most likely arose from duplication. Like its homologue, OXR1, it is capable of reducing the DNA damaging effects of reactive oxygen species when expressed in bacteria, indicating the protein has an activity that can contribute to oxidation resistance. Unlike OXR1, it appears to localize to nuclei and interacts with the estrogen receptor. This raises the possibility that NCOA7 encodes the nuclear counterpart of the mitochondrial OXR1 protein and in mammalian cells it may reduce the oxidative by-products of estrogen metabolite-mediated DNA damage.

## Background

In this study we examine the ability of the nuclear coactivator NCOA7 (formerly called the 140 kDa estrogen receptor associated protein or ERAP140) to function in protection against oxidative DNA damage. Oxidative DNA damage occurs when reactive oxygen species (ROS) attack DNA. ROS are produced as by-products of aerobic metabolism and the damage produced by ROS has been implicated in cancer, neurodegenerative diseases, and aging [1-3].

A number of cellular processes function to prevent the lethal and mutagenic effects of ROS. Protective enzymes fall into two broad categories, those that prevent oxidative DNA damage from occurring and those that repair DNA damage caused by ROS. The damage prevention genes include a wide array of enzymes such as catalases, superoxide dismutases, peroxidases, and thiol containing proteins that detoxify ROS, thereby preventing them from causing damage [4-6]. DNA lesions are produced when ROS escape detoxification and react with, either DNA, or nucleotide pools to produce oxidized bases or sugars. The potential mutagenic effects of oxidized DNA bases are minimized by the DNA repair enzymes [1,7-11]. These DNA repair enzymes include the MutM/Fpg, Ogg1, Nth, and Nei families of glycosylase enzymes that remove oxidized bases from DNA. This group also includes the MutY family which removes A residues that are frequently incorporated opposite the most predominant oxidative lesion, 8-oxoguanine (8-oxoG), during replication [12-15]. A third class of antimutagenic enzymes are the MutT family proteins, which react with oxidized DNA nucleotide triphosphates, 8-oxoG and 8-oxoA, converting them to monophosphates, thereby preventing their incorporation into DNA during replication [16,17].

Imbalances between the normal cellular processes that produce ROS and the mechanisms that prevent and repair oxidative DNA damage can result in increased mutagenesis and cell death [18-20]. Oxidative DNA damage accumulates in cells when an imbalance occurs between ROS production and detoxification. Such an imbalance increases the level of ROS and causes more DNA lesions to be produced than can be processed by the repair enzymes. Increases in oxidative DNA damage can also occur as a result of exposure to exogenous oxidative agents such as ionizing radiation or oxidative chemicals, or a decrease in DNA repair capacity.

The human OXR1 gene was found in a screen for oxidation resistance genes. It is highly conserved, as homologues are found in all sequenced eukaryotic species from yeast to humans [21-24]. OXR1 of yeast and humans is an oxidative and heat stress inducible gene whose product localizes to the mitochondria. When localized to mitochondria of yeast, human OXR1 can complement the peroxide sensitivity of the yeast OXR1 mutant indicating that human OXR1, like its yeast homologue, can function to protect against oxidative DNA damage produced by endogenous and exogenous oxidative agents [21,22]. In this report we characterize a second human gene, called NCOA7, which is highly similar to OXR1. We test its ability to prevent oxidative mutagenesis when expressed in an oxidation dependent mutator strain of Escherichia coli and compare the expression and localization of NCOA7 and OXR1 in human cells.

## Results

### Isolation of NCOA7 and its OXR2 domain

The NCOA7 gene was found in two ways: (1) by searches for estrogen receptor associated protein [25], and (2) by genome searches using the OXR1 protein sequence as a computer probe to search the human genome for DNA sequences potentially capable of encoding OXR1 paralogs [21,22]. The database searches resulted in the identification of four such regions; OXR1 itself, which is located on Chromosome (Chr) 8q23 and an apparent pseudogene on Chr 15 [21]. Two additional regions were found that had the structures consistent with functional genes. One is now called NCOA7 and is located on Chr 6q22.33 and a less conserved gene, tentatively named OXR3, is located on Chr 20q11. Analysis of expressed sequence tag (EST) databases revealed a large collection of ESTs corresponding to OXR1 and NCOA7, suggesting these two genes were expressed. OXR3 was found to correspond to only one EST suggesting it is expressed, either rarely, conditionally, or not at all. Thus we focused this study on the analysis of NCOA7 and compare its properties with those of OXR1.

**Figure 1:**
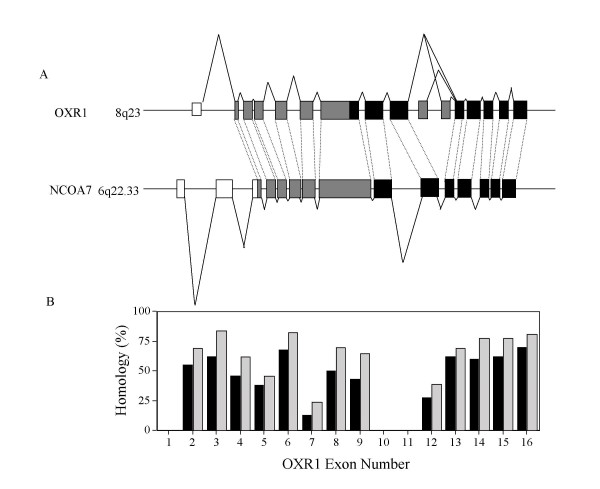
A **Organization of OXR1 and NCOA7 genes**. Exon-intron structures both genes are shown. OXR1 is located on Chromosome 8q22, NCOA7 is located on Chromosome 6q22.33. The black boxes represent exons comprising the minimal OXR domains. Exons shown in gray are those regions that are similar in OXR1 and NCOA7. Areas in white are unique to, either NCOA7, or OXR1. The striped exons are exons 10 and 11, which are also unique to OXR1. The length of the lines connecting exons is an indication of the relative size of the intron. B. Shows the comparison of the extent of identity of individual exons (black boxes) and similarity (gray boxes). The exon numbers listed are those of OXR1.

Figure 1A compares all of the known protein coding exons of OXR1 and NCOA7. The similarity between the two genes is extensive and genomic analysis indicates a similar gene structure that includes retention of exon boundaries, suggesting they share a common origin and are likely to have arisen from a duplication event. Figure 1A also shows, in black, the OXR domain cDNA of NCOA7 that comprises Image clone 608928 and compares it with the form of the OXR1 gene previously described (also in black) [21,22]. The overall identity between full length OXR1 and NCOA7 is 38%. An overall similarity of 53% is calculated using standard BLAST parameters allowing conservative substitutions [26] and correcting for computer generated truncations of non similar ends. However specific regions are considerably more highly conserved and others are unique to NCOA7 or OXR1. Figure 1B compares the extent of identity of individual exons. The upstream exons of NCOA7 are unique and are not represented in the DNA upstream of OXR1, as no sequences capable of encoding a related peptide are present on Chr 8 upstream of OXR1. Conversely, there is no sequence present on Chr 6 in the genomic region upstream of NCOA7 that is similar to the first exon of OXR1. Thus, the upstream exons indicated as unfilled boxes in Figure 1A represent regions that are unique to either NCOA7 or OXR1. Analysis of the Chr 6 DNA sequence of the NCOA7 coding region also failed to detect the presence of DNA sequences capable of encoding peptides related to those encoded by exons 10 and 11 of OXR1, ruling out the possibility of potential NCOA7 splice variants that contain exons related to these two exons of OXR1.

### NCOA7 can protect cells from oxidative DNA damage

**Figure 2:**
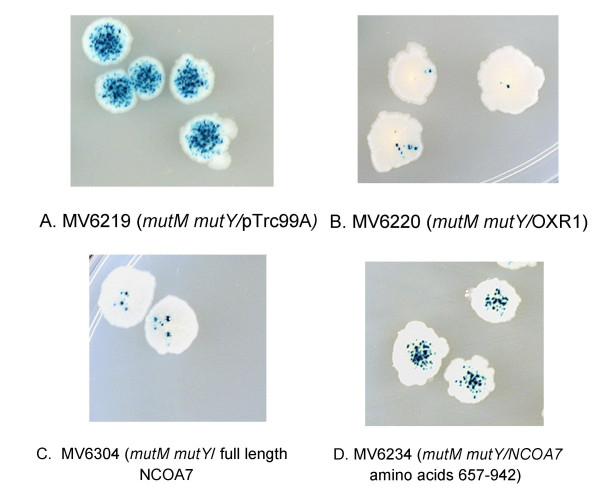
**Bacterial Papillation Assay**. Individual colonies of the white *E. coli lacZ *cc104 mutant colonies containing the dark blue microcolonies which are the GC→TA revertants. Panel A shows the high spontaneous mutation frequency of the *mutM mutY *strain carrying only the vector. The remaining panels show the reduction in LacZ papillation in the *mutM mutY *strain resulting from the expression of either full length, (B) OXR1C; (C), full length NCOA7; (D), NCOA7 (657–942).

In order to determine if the highly conserved OXR1 and NCOA7 are also functionally related, we performed experiments similar to those that led to the isolation of OXR1 and demonstration of its ability to protect cells from oxidative DNA damage [21,22]. The protection of cells from oxidative DNA damage by human OXR1 was most clearly demonstrated using an *mutM mutY *mutant strain of *E. coli*. The combination of these two mutations causes a synergistic increase in GC→TA transversion mutagenesis due to the bacterial cell's inability to prevent mutagenesis by 8-oxoguanine (8-oxoG), the predominant oxidative DNA lesion [11]. MutM is required for the removal of 8-oxoG and MutY is required for the removal of A mispaired with 8-oxoG, the predominant replication intermediate leading to mutagenesis by 8-oxoG. Since 8-oxoG lesions result in GC→TA transversion, the level of oxidative DNA damage can be monitored using the *lacZ *cc104 allele. This allele reverts to Lac+ only by GC→TA transversion [11] and this transversion is produced primarily as a result of lesions repairable by the *E. coli *MutM 8-oxoG DNA glycosylase enzyme [27,28]. 8-oxoG lesions result from the spontaneous production of ROS as a by-product of normal aerobic metabolism, which in turn reacts with DNA, producing lesions that give rise to mutations. The cells inability to repair the lesions, or to remove A mispaired with 8-oxoG results in a mutator phenotype (Figure 2A).

Since expression of the human OXR1 cDNA in the *mutM mutY *mutator strain of *E. coli *results in suppression of spontaneous oxidative GC→TA transversion mutagenesis [21,22], we tested if expression of NCOA7 produces a similar antimutator activity. The full length NCOA7 cDNA was transferred to the pTrc99a vector and introduced into the *mutM mutY *strain. Figure 2C shows that this clone essentially abolishes spontaneous oxidative mutagenesis. This indicates that the full length NCOA7 protein functions to protect cells against oxidative DNA damage when expressed in *E. coli*. Quantitative mutagenesis assays confirm the ability of full length NCOA7 to suppress GC→TA transversion mutagenesis and demonstrate that oxidative mutagenesis is reduced by more than 99.9%, which is similar to the spontaneous levels of mutagenesis seen in a wild type, repair proficient strain of *E. coli *(Table 1).

**Table 1:** Quantitative mutation suppression.

Strain genotype	Plasmid/insert	Mutation frequency^a^	Standard deviation	Mutagenesis suppression (% reduction of vector control)
*mutM mutY*	pTrc99a vector only	9799	± 2848	NA^b^
*mutM mutY*	pTrc99a/OXR1 [21]	773	± 324	* 90
*mutM mutY*	pTrc99a/NCOA7	0.26	± 0.22	* >99.9
*mutM mutY*	pTrc99a/OXR2 domain of NCOA7 (657–942)	2635	± 725	* 73
Wild type	pTrc99a vector	0.31	± 0.17	* NA

^a^Mutation frequency (Mutants/10^7 ^viable cells). Data represent the averages of at least 6 independent measurements.

^b ^Not Applicable.

**Figure 3:**
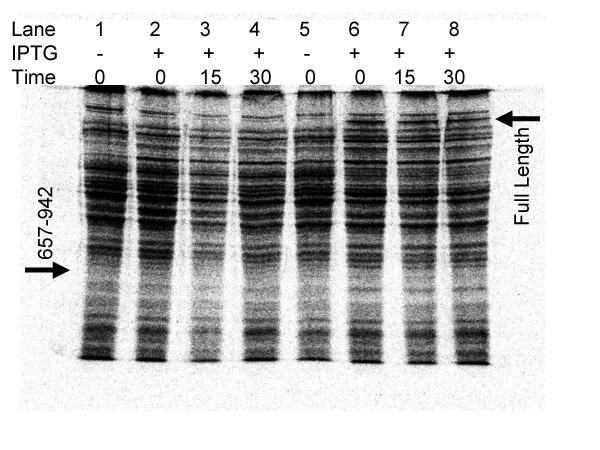
**Expression and stability of full length and truncated NCOA7 proteins in *E. coli***. Cells were either induced or not with IPTG. Proteins from induced or uninduced exponential phase cells were labeled with 35 [S] Met and chased, then harvested either immediately, or after 15, or 30 min further incubation as indicated in the figure. The arrows indicate the positions of the full length and truncated (657–942) forms of NCOA7 protein.

In the case of the OXR1, expression of the short OXR1C isoform, shown in black in Figure 1, is sufficient for its antimutator function in the bacterial assay [21,22]. To test if the oxidation resistance activity of NCOA7 coding sequences also lie in the corresponding region we constructed clones that lacked upstream regions and produced truncated NCOA7 proteins similar to OXR1C. The NCOA7 (657–942) clone begins at amino acid residue 657 of NCOA7 and extends to its normal termination codon [25]. Expression of this clone reduces the oxidative mutator phenotype by 73% when expressed the *E. coli mutM mutY *mutant strain (Figure 2D and Table 1). To test if the weak activity of the truncated protein is due to lower levels of expression, or instability of the protein, we pulse labeled total cellular proteins of IPTG induced and uninduced cells with ^35^S-methionine, prepared extracts at various times after labeling and separated the proteins on 12% polyacrylamide gels by electrophoresis, then scanned for IPTG inducible bands of the expected molecular weights immediately after labeling and after 15 and 30 minutes of further incubation. The full length NCOA7 protein was readily apparent as a strong band migrating at the expected molecular weight of 106 kDa, based on the primary amino acid sequence. It appears to be relatively stable, showing no detectable diminution in intensity upon further incubation (Figure 3). The 657–942 fragment is seen as a faint band at its expected molecular weight of approximately 33 kDa (Lane 2, Figure 3). It appears to be relatively unstable, since it is weakly detectable only at the initial time point immediately after the 5 min chase with cold methionine, and is no longer detectable after 15 and 30 min further incubation (Lanes 3 and 4, Figure 3). Thus the weak activity of the 657–942 fragment in the mutagenesis assay is most likely due to its apparent instability. Despite the low level of expression of the C-terminal domain compared to the full length protein, the truncated protein is still capable of suppressing 73% of the oxidative mutagenesis. Thus we propose that C-terminal domain of NCOA7 and OXR1 proteins defines the OXR domain that protects cells from oxidative mutagenesis.

### NCOA7 is localized to the nucleus

**Figure 4:**
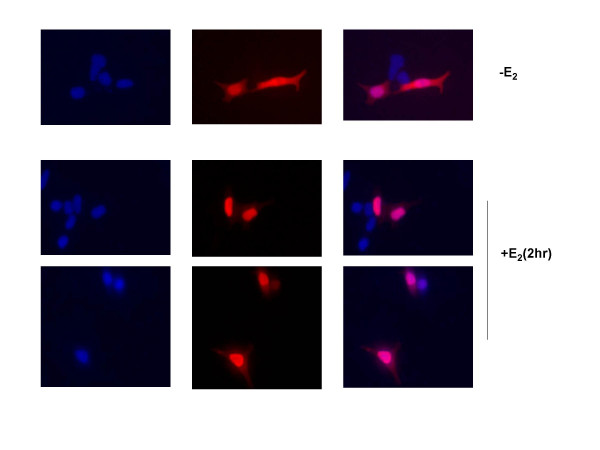
**Subcellular localization of full length FLAG-tagged NCOA7 protein in human MCF-7 cells**. MCF-7 cells were cultured in hormone-free medium and transiently transfected with FLAG-tagged full-length NCOA7. Two days after transfection, cells were treated without or with 100 nM E2 for 2 hours. Cellular localization of NCOA7 was detected by immunofluorescence using an anti-FLAG antibody (red stain). Cell nuclei were indicated by the blue DAPI stain. -E2, no estrogen, +E2, estrogen treated cells.

Protein sequence analysis of NCOA7 indicated that it has a putative nuclear localization signal. In order to determine if this sequence does in fact direct the protein to the nucleus, we produced a FLAG tagged form of NCOA7 and expressed it in MCF-7 cells by transient transfection. NCOA7 was originally identified as an estrogen receptor-associated protein. To examine whether its cellular localization may be affected by estrogen stimulation, cells were grown in the absence of hormone and then treated with 100 nM 17 β-estradiol (E2) for 2 hrs. Figure 4 shows that in the absence of hormone, NCOA7 exhibits a localization that is both cytoplasmic and nuclear. The cytoplasmic localization of NCOA7 differs from that of OXR1, which shows a punctate pattern of staining that colocalizes with the mitochondrial marker Mitotracker indicating its mitochondrial localization [22]. While we can not rule out the possibility that NCOA7 is present in mitochondria, it differs from OXR1 and is clearly not concentrated in this organelle. Therefore OXR1 and NCOA7 show different localization properties. OXR1 is excluded from nuclei and localizes to mitochondria, whereas NCOA7 shows diffuse cytoplasmic staining and localizes to nuclei (Figure 4). Upon treatment of cells with estradiol (E2), NCOA7 is concentrated in the nucleus and cytoplasmic staining appears to be reduced. Thus the treatment with E2 appears to stimulate nuclear localization.

### NCOA7 is not induced by peroxide treatment

**Figure 5:**
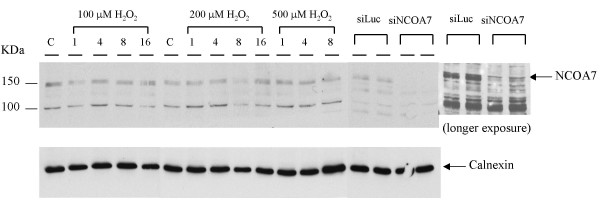
**Protein expression of NCOA7 after treatment with hydrogen peroxide**. MCF-7 cells were treated with indicated concentrations of hydrogen peroxide (H_2_O_2_) for 1, 4, 8, or 16 hours. Whole cell lysates were prepared for western analysis. The protein band corresponding to NCOA7 was indicated by its loss after siRNA-mediated inhibition. Calnexin is a loading control.

OXR1 and many other proteins that protect against oxidative DNA damage are inducible upon exposure to hydrogen peroxide [22]. In order to determine if NCOA7 is induced in response to peroxide treatments, MCF-7 cells were treated with hydrogen peroxide, proteins extracted at various times post treatment. NCOA7 levels were then measured by western blot. Figure 5 shows that only the 140 kDa band is reduced by NCOA7 specific siRNA treatments, indicating that this is the NCOA7 band. Examination of peroxide treated cells shows that the levels of NCOA7 protein are not detectably altered in response to treatment. Thus we conclude that NCOA7 is constitutively expressed and is not induced by peroxide treatment, differing in this respect from OXR1.

## Discussion

Comparisons of the OXR gene family indicate several key events have occurred during evolution of OXR domain proteins. *S. cerevisiae *carries only one copy of OXR in its genome. It is 273 amino acids in length and includes only sequences corresponding to the C-terminal OXR domains of NCOA7 and OXR1. In higher organisms, the OXR domain has become associated with additional upstream protein coding sequences. This occurred prior to duplication, since there is a high degree of identity and similarity between NCOA7 and OXR1 throughout their sequences. The exceptions to this are their N termini, which, in NCOA7 contains a nuclear localization sequence, which is absent in the mitochondrially targeted OXR1. Portions of their largest central exons are also dissimilar. In NCOA7 its exon 8 is 357 amino acids in length and contains its estrogen receptor binding site [25], whereas the corresponding exon 7 of OXR1 is only 255 amino acids in length and lacks the estrogen receptor binding sequences. OXR1 also contains several unique exons. These include exon 10, which has a readily recognizable mitochondrial targeting sequence [22], and exon 11, which is found in only one OXR1 isoform (Fig. 1).

The demonstration that the full length NCOA7 protein can function to prevent oxidative mutagenesis when expressed in bacteria suggests it may function in this manner in its native eukaryotic host. In bacteria, this may be a general function that results in detoxification of various ROS molecules. The key role for the C-terminal OXR domains in oxidation resistance is indicated by (1) the oxidation sensitivity resulting from deletion of the OXR1 gene of yeast [21]; (2) the ability of mitochondrially targeted human OXR domain of OXR1 to complement the H_2_O_2 _sensitivity of the yeast *oxr1 *deletion mutant [22]; and (3) the ability of the OXR domains of either OXR1 or NCOA7 to suppress the oxidative mutator phenotype of oxidation sensitive *E. coli *mutants [22] (and Figure 2). Thus we refer to the C-terminal region of NCOA7 and OXR1 as the oxidation resistance, or OXR domain. Comparison of the OXR domains of OXR1 and NCOA7 with the yeast gene product, indicates both human genes are approximately equally similar to the yeast protein when their OXR domains are compared with the full length yeast protein; OXR1 has 27%identity and 44% similarity to yeast OXR1 and NCOA7 has 31% identity and 43% similarity. Although both human genes are equally similar to the single *S. cerevisiae *OXR gene, the yeast OXR gene is functionally most similar to human OXR1, since both yeast and human OXR1 proteins are induced by hydrogen peroxide and heat stress, and localize to mitochondria [22].

The association of the NCOA7 gene product with the estrogen receptor is curious for a gene product involved in protection from oxidative DNA damage. It is noteworthy that several DNA repair proteins have recently been identified as estrogen receptor associated proteins. These include the O^6^-methylguanine methyltransferase DNA repair protein, the 3-methyladenine DNA N-glycosylase repair protein, and the TG specific mismatch repair protein TDG [29-31]. The result that NCOA7 is another ER associated protein that has DNA maintenance properties, suggests that ER association of these related classes of proteins may be a common feature. It has been proposed that NCOA7 may sense the oxidative state of the cell and regulate responses to oxidative DNA damage and the result that NCOA7 can function to protect cells from oxidative DNA damage strengthens this hypothesis [25]. It may also play a direct role in oxidation resistance, a possibility that is particularly intriguing in light of results indicating that estrogen metabolism causes oxidative DNA damage (for review see: [32]). When estrogens, such as β-estradiol, are metabolized to catechol estrogen quinones and semiquinones, they enter into a redox cycling reaction in which the quinones are reduced to semiquinones. The semiquinones, in turn, spontaneously oxidize to back to quinones producing ROS [33]. Oxidative DNA damage has been demonstrated to result as a by-product of estradiol metabolism [34], thus it is possible that NCOA7 functions to mitigate oxidative DNA damage resulting from estrogen metabolism by bringing it in close proximity to estrogens upon import into the nucleus. Moreover, such an oxidation resistance mechanism of NCOA7 should be enhanced by the presence of estrogen, since this stimulates NCOA7 entry into the nucleus (Figure 4).

Both NCOA7 and OXR1 gene products show their highest levels of expression in brain tissue [22,25], suggesting they may play a critical role in protecting brain cells from oxidative DNA damage. Thus, it will be of interest to see if either or both of these proteins function to protect against neurodegenerative diseases that are affected by oxidative damage and apoptosis.

## Conclusion

The NCOA7 gene produces a product that is similar to OXR1 in sequence and in function. It is able to increase resistance to prevent oxidative mutagenesis when expressed in bacteria. This function requires only its C-terminal OXR domain, which is conserved from yeast to human cells. NCOA7 differs from OXR1 in several key respects, unlike the mitochondrial and inducible OXR1 gene product, the NCOA7 gene product localizes to the nucleus and is associated with the estrogen receptor. Thus, these two oxidation resistance proteins appear to have different and unique roles. Yeast carries only a small OXR1-like protein that is similar to the OXR domains of both OXR1 and NCOA7, but is functionally most similar to mammalian OXR1. In higher eukaryotes the two OXR domain genes appear to have arisen by duplication of an ancestral OXR gene after acquiring a common upstream sequence.

## Methods

### Construction of NCOA7 and OXR2 domain expression vectors

Potential OXR protein coding regions were identified by searches of the human genome using the OXR1 protein sequence described previously [21] as a computer probe using the tBLASTn program to scan the human genome [26]. The potential OXR1 coding sequences identified were then used to find corresponding expressed sequence tags (ESTs). cDNA clones expressing the ESTs were from the IMAGE consortium clone bank and obtained either from In Vitrogen (Carlsbad, CA) or Clonetech/BD-Bioscience (Mountainview, CA), then sequenced to confirm their identity. One cDNA, image clone 608928, carries the sequences of region of chromosome 6q22.33 that are similar to the OXR1C isoform sequence of OXR1 described previously [21]. Digestion of this clone with EcoR1 and Xho1 released the cDNA region and allowed its transfer to the prokaryotic expression vector pTrc99a (Pharmacia). This domain of NCOA7 was subcloned from 608928 by PCR using primers EcoRI-up-608928 (ATC ATC GAA TTC AAA GAA GAA AAA AGC AAG) and SalI-down (ATC ATC GTC GAC ATC AAA TGC CCA CAC CTC) then digesting the PCR products with EcoR1 and Sal1 and inserting the digested PCR product into the EcoR1 and Sal1 sites of the pTrc99A expression vector to produce NCOA7 (657–942). The full length NCOA7 cDNA was transferred from the pcDNA 3.1 vector [25] to the pTrc99A bacterial expression vector by digestion with BamHI and XhoI and ligating the 5 kb NCOA7 fragment into the BamHI and SalI sites of the pTrc99A vector.

### Mutagenesis assays

Mutagenesis assays were performed essentially as described elsewhere [28]. Briefly, full length NCOA7, or OXR domain coding regions were expressed from the pTrc99A vector in a *mutM mutY *strain of *E. coli*. This strain carries the *lacZ *cc104 allele which reverts only by GC→TA transversion [11], a signature mutation of oxidative DNA damage[27,28]. Mutagenesis is assessed as the number of dark blue, LacZ+ revertant papillae that appear within individual white LacZ-colonies after 5 days incubation. Quantitative mutagenesis assays were performed by growing cells overnight, spreading cells on plates that contain lactose as the sole carbon source to determine the number of Lac+ revertants, and on glucose plates to determine the total number of cells. LacZ reversion frequencies are expressed as revertants/10^7 ^viable cells.

### Protein expression in *E. coli*

Cells were grown to early exponential phase (approx. 10^7 ^cells/ml), induced with 1 mM IPTG for 90 min, or uninduced, then pulse labeled with ^35 ^[S]-Met (10 μCi/ml) for 5 min, chased with 100 μg/ml cold Met for 5 min, then harvested immediately (lanes 1, 2, 5 and 6), incubated for an additional 15 min (lanes 3 and 7), or 30 min (lanes 4 and 8) in order to assess protein stability. Protein extracts were prepared for separation on 12% SDS-polyacrylamide gel electrophoresis using standard methods described elsewhere[35]. Gels were analyzed using a Fuji BAS-2500 phosphorimager and accompanying Fuji Film image analysis software.

### Immunofluorescence Assay

FLAG-tagged full-length NCOA7 was transiently transfected into a breast cancer cell line MCF-7 following the manufacturer's protocol (Lipofectamine 2000, Invitrogen). To investigate estradiol-dependent localization of NCOA7, cells were maintained under hormone-free conditions. Two days post transfection, cells were treated without or with 100 nM 17 β-estradiol (E2) for 2 hours. Following treatment, cells were washed with Phosphate Buffered Saline (PBS) and fixed in 3.7% formaldehyde in PBS for 10 min at 40°C. Cells were then washed with PBS and permeabilized with 0.2% Triton X-100 for 5 min at 40C. Cells were blocked in 10% fetal bovine serum (FBS) for 30 min at room temperature, and incubated with M5 anti-FLAG antibody (Sigma) at 1:500 dilution in 5% FBS for 1 hr at room temperature. After the primary antibody incubation, cells were washed with PBS and incubated with secondary antibody (AlexaFluor-568 goat anti-mouse, Molecular Probes) at 1:1000 dilution for 45 min. After washing in PBS, cells were mounted onto slides with Vectashield containing DAPI and imaged by fluorescence microscopy.

### Western analysis

MCF-7 cells were treated without or with varying concentrations of hydrogen peroxide (H_2_O_2_) for 1, 4, 8, or 16 hours. Whole-cell lysates were then prepared in RIPA lysis buffer (0.15 mM NaCl/0.05 mM Tris·HCl, pH 7.2/1% Triton X-100/1% sodium deoxycholate/0.1% SDS). 40 μg of the lysates was resolved by SDS/PAGE, transferred to a nitrocellulose membrane, and blotted with an anti-NCOA7 antibody. Cell lysates in which the NCOA7 expression was inhibited by siRNA targeting NCOA7 were included to identify the protein band corresponding NCOA7.

## Authors' contributions

MD produced bacterial vectors that expressed NCOA7 and conducted most the antimutator assays, AK conducted additional antimutator studies and produced vector expressing full length NCOA7 in bacteria, TF cloned and characterized the OXR domains, NE supervised and contributed to all of the above studies and conducted data analysis, WS and MB conducted the experiments with eukaryotic cells, MV, conducted the protein stability studies, the computer analyses and drafted the manuscript.
